# Supplemental oxygen therapy use among patients with fibrosing interstitial lung disease in the United States

**DOI:** 10.1186/s12931-025-03139-3

**Published:** 2025-02-28

**Authors:** Joseph Yang, Andrea Steffens, Amy L. Olson, Amy Anderson, Gursimran Basra, Phani Veeranki, Joao A. de Andrade

**Affiliations:** 1 Boehringer Ingelheim Pharmaceuticals, Inc., Ridgefield, CT USA; 2 Optum, Eden Prairie, MN USA; 3 Vanderbilt University Medical Center, Nashville, TN USA

**Keywords:** Idiopathic pulmonary fibrosis, Fibrosing interstitial lung disease, Supplemental oxygen therapy, Non-IPF interstitial lung disease

## Abstract

**Background:**

Supplemental oxygen therapy is commonly prescribed in clinical practice for patients with fibrosing interstitial lung disease (ILD) to reduce breathlessness and increase physical capacity. Only a few studies have evaluated the incidence of oxygen therapy use, with evidence lacking in its use among fibrosing ILD subtypes including patients with idiopathic pulmonary fibrosis (IPF) and non-IPF ILD. This study aimed to estimate incidence of oxygen therapy and factors associated with oxygen therapy initiation.

**Methods:**

This non-interventional study used US administrative claims and electronic health record data from 01 October 2015 to 30 June 2022. Patients aged ≥ 18 years with newly diagnosed fibrosing ILD (≥ 2 fibrosing ILD diagnoses in any position on different dates of service within 365 days) were included; the index date was the first date with ILD diagnosis. Patients were followed until the earlier of health plan disenrollment, death, or end of study period. Oxygen therapy use was evaluated among patients without evidence of oxygen therapy before the index date, stratified by the underlying fibrosing disease (i.e., IPF vs. non-IPF ILD). Factors associated with oxygen therapy use were evaluated using Cox proportional hazards regression.

**Results:**

A total of 114,921 patients (IPF *n* = 5,555; non-IPF ILD *n* = 109,366) newly diagnosed with fibrosing ILD were included in the study. The mean (standard deviation) age of patients with ILD was 66.9 (14.2) years, and 47.2% were male. Patients were followed for a mean of 24 months after ILD diagnosis, during which 38% of fibrosing ILD patients initiated oxygen therapy; a higher proportion of patients with IPF initiated oxygen therapy compared to those with non-IPF ILD (68% and 36%, respectively). Factors associated with oxygen therapy initiation included IPF, higher Charlson comorbidity scores, and comorbidities that impair respiratory capacity.

**Conclusions:**

The study findings demonstrate a substantial proportion of patients with fibrosing ILD initiated oxygen therapy following initial ILD diagnosis, with higher rates of oxygen therapy initiation observed among patients with IPF compared with non-IPF ILD. Respiratory comorbidities were key factors associated with increased initiation of oxygen therapy.

**Supplementary Information:**

The online version contains supplementary material available at 10.1186/s12931-025-03139-3.

## Introduction

Interstitial lung disease (ILD) is a term that describes a heterogeneous group of approximately 200 conditions that are characterized by varying degrees of inflammation and fibrosis of the lung parenchyma. Common clinical manifestations include dyspnea and exertional limitation and persistent cough, with the eventual development of hypoxemia, respiratory failure, and death [1, 2]. 

Disease modifying treatment options for these patients are limited. Antifibrotic agents (i.e., nintedanib and pirfenidone) reduce the rate of lung function decline in patients with idiopathic pulmonary fibrosis (IPF), and nintedanib was found to have a similar effect amongst patients with non-IPF progressive pulmonary fibrosis (PPF) [3]. Immunosuppression is often used to treat patients with certain forms of PPF, such as those related to connective tissue diseases and hypersensitivity pneumonitis [4]. Despite the therapies currently available, many patients continue to experience progressive clinical deterioration with increasing symptom burden and loss of quality of life [5, 6]. Multidisciplinary and collaborative support is recommended to patients with fibrosing ILD throughout their disease course, aiming to relieve symptoms, preserve functional capacity, and maintain quality of life [7]. Additional management strategies that are often employed include pulmonary rehabilitation, patient education, peer support groups, and supplemental oxygen therapy [2, 8–10]. 

Analysis of registry populations suggest that hypoxemia is an independent risk factor for poor outcomes in pulmonary fibrosis, and supplemental oxygen therapy is often prescribed for patients with ILD [11]. Unfortunately, the evidence supporting the use of supplemental oxygen therapy in ILD patients is largely derived from clinical trials that demonstrated improved outcomes in patients with chronic obstructive pulmonary disease (COPD). This is a population that differs markedly from patients with fibrosing ILD regarding the physiology of exercise limitation and the magnitude of exertional hypoxemia [2, 12, 13]. Given the severity of exertional hypoxemia that has been shown to be increased among patients with ILD compared to those with COPD, the effectiveness of oxygen therapy in the treatment of ILD is unclear [14]. A number of studies have demonstrated that long-term oxygen therapy may improve exercise capacity and health-related quality of life for ILD patients [15], however, the data are less conclusive regarding the reduction of dyspnea and survival [10, 16]. Additionally, oxygen therapy imposes logistical and financial burdens that could offset potential long-term benefits [17]. 

With limited literature describing the use of oxygen therapy among fibrosing ILD patients in a real-world setting, our study aimed to close the knowledge gap by estimating the time to oxygen therapy initiation among patients with newly diagnosed fibrotic ILD and evaluating factors associated with oxygen therapy initiation.

## Methods

### Data sources

This was a retrospective study that used existing US administrative claims and electronic health record (EHR) data from Optum’s de-identified Market Clarity data from 01 October 2015 through 30 June 2022 (study period). Optum^®^ Market Clarity is an integrated, multi-source medical claims, pharmacy claims, and electronic health records data set that links EHR data - including lab results, vital signs and measurements, diagnoses, procedures and information derived from unstructured clinical notes using natural language processing - with historical, linked administrative claim data - including pharmacy claims, physician claims, clinical information facility claims and medications prescribed and administered. Optum^®^ Market Clarity is statistically de-identified under the HIPAA Privacy Rule’s Expert Determination method and managed according to Optum^®^ customer data use agreements [11, 12]. 

### Study population

The study population comprised adults aged ≥ 18 years with newly diagnosed fibrosing ILD identified from 01 October 2016 through 30 June 2022 (patient identification period). Patients were identified using International Classification of Disease, Tenth Revision, Clinical Modification (ICD-10-CM) diagnosis codes. A patient with newly diagnosed fibrosing ILD was defined as having at least two separate encounters with fibrosing ILD diagnoses on different dates of service and within 365 days of each other. The two fibrosing ILD diagnoses could be one of the following combinations: at least two encounters with fibrosis codes (i.e., ICD-10-CM codes specific to lung fibrosis), or at least one encounter with a fibrosis code and one encounter with the underlying diseases that can lead to lung fibrosis. Patients who met the initial selection criteria were categorized into groups based on the known causes of fibrosis (i.e., idiopathic pulmonary fibrosis [IPF] or non-IPF ILD). The first ILD diagnosis date was set as the index date, and patients were followed until the earlier of disenrolling in either medical or pharmacy benefits, death, or end of study period. Patients were required to have at least 12 months of continuous insurance enrollment prior to the index date to collect patient demographics and clinical characteristics (pre-ILD baseline period). Patients were excluded if they had a fibrosing ILD diagnosis in the pre-ILD baseline period, or if their age, gender, or geographic region was unknown.

### Study measures

#### Demographic and clinical characteristics

All demographic and clinical characteristics were measured during the pre-ILD baseline period. Patient demographic and clinical characteristics included age as of the index year, gender, race, insurance type, US Census region, as well as the Quan-Charlson comorbidity index score (CCI) and respiratory comorbidities, based on diagnosis codes on medical claims [18, 19]. Lastly, provider specialty (primary care physician [including general/family practice, internal medicine], pulmonology and rheumatology), medication use (corticosteroid [methylprednisolone, prednisone, prednisolone], biologics [abatacept, denosumab, etanercept, tocilizumab, infliximab, rituximab], calcineurin inhibitors [cyclosporine, tacrolimus], and other immunosuppressants [mycophenolate mofetil, hydroxychloroquine, leflunomide, methotrexate, sulfasalazine, chloroquine phosphate]), all-cause healthcare resource utilization (HCRU) (ambulatory visits [physician office and hospital outpatient], emergency department (ER) visits, inpatient admissions, and pharmacy fills) and healthcare costs were also assessed during the pre-ILD baseline period.

### Outcomes

The primary outcomes included the incidence rate of oxygen therapy initiation and time to oxygen therapy initiation, which was defined as the duration in months from the index date to the first evidence of oxygen use, among patients without oxygen therapy use during the pre-ILD baseline period. Sustained oxygen therapy use, defined as the presence of ≥ 11 claims for oxygen therapy in a 12-month period, was measured among patients with at least 12 months of follow-up after the initial oxygen therapy claim [13]. Evidence of oxygen use was determined by the presence of ≥ 1 diagnosis (ICD-10-CM), procedure (HCPCS, CPT), or revenue code for oxygen therapy (Supplementary Table S1).

### Statistical analysis

All variables were analyzed descriptively, and results were reported overall and stratified by IPF vs. non-IPF ILD. Categorical variables were reported as numbers and percentages; continuous variables were reported as mean (standard deviations [SD]) and median (interquartile range [IQR]). The two-sample t-test was used to assess continuous measures, and Pearson chi-square test was used assess categorical measures. Incidence of oxygen therapy was calculated as the total number of patients with ILD who initiated oxygen therapy divided by the sum of follow-up time at risk (in person-years) for all patients with ILD. Kaplan–Meier (KM) analysis was used to evaluate the censor-adjusted time to oxygen therapy initiation from the index date. Censoring occurred at the first of the following events: disenrollment from the health plan, end of the study period, or death. Based on the KM analysis, the cumulative incidence function was plotted. Cox proportional hazards regression was used to identify factors associated with initiation of oxygen therapy. Confidence intervals (CI) and p-values were also reported for all covariates in the model. Analysis was conducted using SAS Software, Version 9.4 [14]. 

## Results

### Pre-ILD baseline demographic and clinical characteristics

**Fig. 1:**
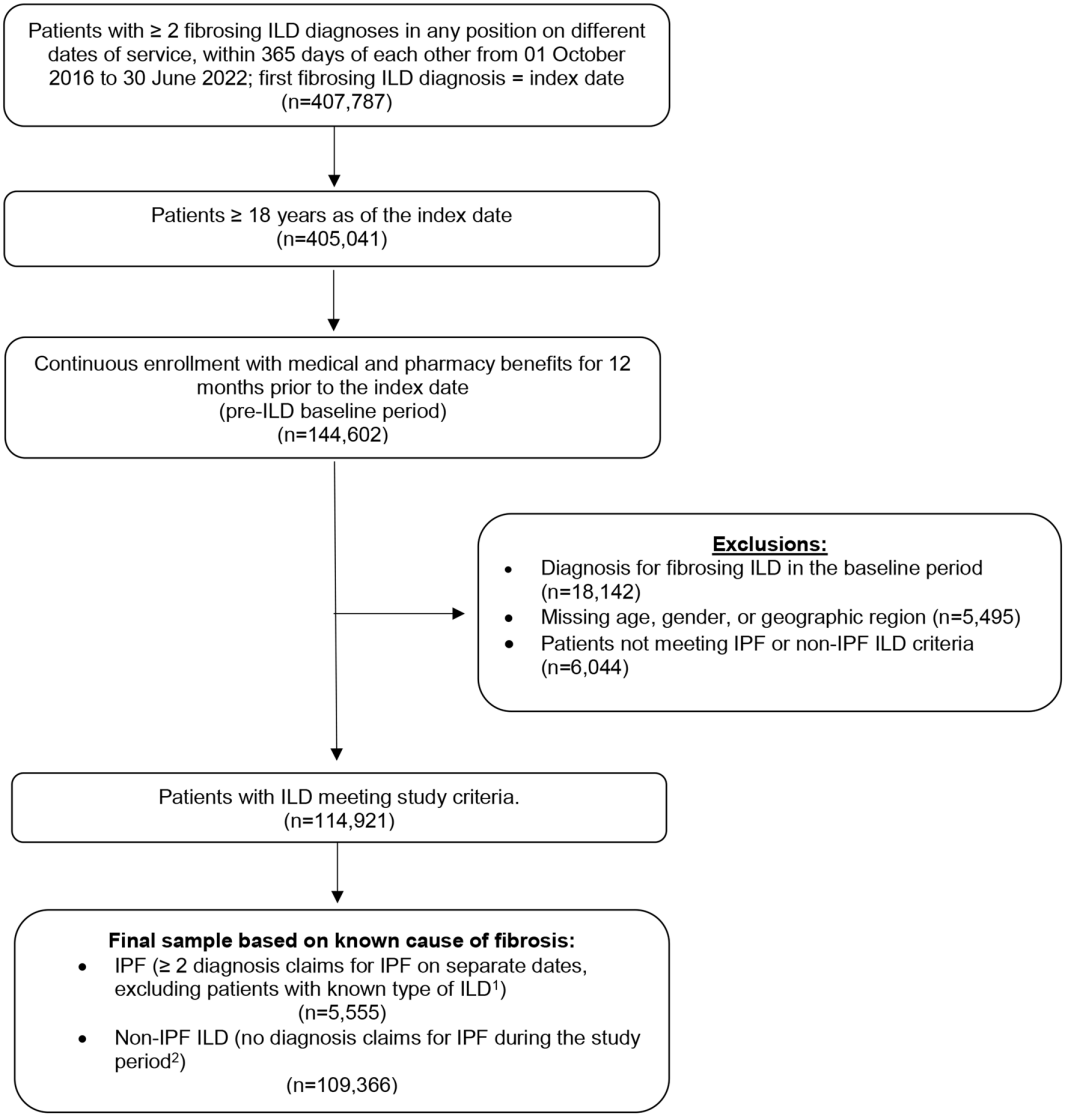
Patient sample selection. ILD = interstitial lung disease; IPF = idiopathic pulmonary fibrosis. ^1^Defined as at least 1 claim with diagnosis for autoimmune ILD, hypersensitivity pneumonitis, or sarcoidosis during the study period. ^2^Patients with just 1 claim with diagnosis for ILD were excluded from analysis

A total of 114,921 patients with ILD were included in the final study sample; of these, 5,555 patients (6.9%) were included in the IPF group and 109,366 (93.1%) were included in the non-IPF ILD group (Fig. 1). The mean (SD) age of the overall sample was 66.9 (14.2) years, 52.8% were female, and 67.7% were White (Table 1). Patients were predominantly from the Midwest (40.5%), followed by the South (27.6%); 43.1% had Medicare insurance coverage. The mean (SD) baseline CCI score among patients with ILD was 2.5 (2.4), and the distribution of underlying ILD types was 17.0% hypersensitivity pneumonitis (HP), 15.5% autoimmune ILD, 3.3% sarcoidosis, 3.6% multiple, and 60.6% unclassified. Over one-third of patients had baseline corticosteroid medication use (38.0%), and COPD was the most observed respiratory comorbidity (35.2%). Oxygen therapy use during the pre-ILD baseline period was observed among 18.6% of patients with ILD. Additional baseline clinical characteristics can be found in Table 1. Baseline HCRU and healthcare costs were measured and can be found in Supplementary Table S2.

**Table 1:** Pre-ILD baseline demographic and clinical characteristics

Demographics	Total (*N* = 114,921)	IPF (*N* = 5,555)	Non-IPF ILD (*N* = 109,366)	*p*-value
Age (continuous), mean (SD)	66.9 (14.2)	72.4 (10.4)	66.7 (14.3)	< 0.001
Gender, n (%)				< 0.001
Female	60,669 (52.8)	2,167 (39.0)	58,502 (53.5)	
Male	54,252 (47.2)	3,388 (61.0)	50,864 (46.5)	
US Region^1^, n (%)				
Northeast	25,815 (22.5)	1,326 (23.9)	24,489 (22.4)	0.010
Midwest	46,507 (40.5)	2,145 (38.6)	44,362 (40.6)	0.004
South	31,701 (27.6)	1,509 (27.2)	30,192 (27.6)	0.472
West	10,898 (9.5)	575 (10.4)	10,323 (9.4)	0.024
Race, n (%)				
White	77,744 (67.7)	4,073 (73.3)	73,671 (67.4)	< 0.001
African American	11,492 (10.0)	274 (4.9)	11,218 (10.3)	< 0.001
Asian	1,824 (1.6)	113 (2.0)	1,711 (1.6)	0.006
Unknown	23,861 (20.8)	1,095 (19.7)	22,766 (20.8)	0.048
Ethnicity, n (%)				
Hispanic	5,455 (4.8)	227 (4.1)	5,228 (4.8)	0.018
Not Hispanic	78,150 (68.0)	3,825 (68.9)	74,325 (68.0)	0.162
Unknown	31,316 (27.3)	1,503 (27.1)	29,813 (27.3)	0.740
Insurance type, n (%)				
Commercial only	39,533 (34.4)	1,718 (30.9)	37,815 (34.6)	< 0.001
Medicare only	49,475 (43.1)	2,825 (50.9)	46,650 (42.7)	< 0.001
Medicaid only	12,420 (10.8)	269 (4.8)	12,151 (11.1)	< 0.001
Multiple known types	10,194 (8.9)	552 (9.9)	9,642 (8.8)	0.004
Unknown type	3,299 (2.9)	191 (3.4)	3,108 (2.8)	0.009
Baseline Charlson comorbidity score (continuous), mean (SD)	2.5 (2.4)	1.8 (1.9)	2.5 (2.5)	< 0.001
Baseline Charlson comorbidity score (categorical), n (%)				
0	26,689 (23.2)	1,723 (31.0)	24,966 (22.8)	< 0.001
1–2	40,841 (35.5)	2,196 (39.5)	38,645 (35.3)	< 0.001
3–4	25,885 (22.5)	1,123 (20.2)	24,762 (22.6)	< 0.001
5+	21,506 (18.7)	513 (9.2)	20,993 (19.2)	< 0.001
Underlying ILD type, n (%)				
Autoimmune ILD	17,827 (15.5)	0 (0)	17,827 (16.3)	
Hypersensitivity pneumonitis	19,570 (17.0)	0 (0)	19,570 (17.9)	
Sarcoidosis	3,801 (3.3)	0 (0)	3,801 (3.5)	
Multiple known types	4,094 (3.6)	0 (0)	4,094 (3.7)	
Unclassified	69,629 (60.6)	5,555 (100)	64,074 (58.6)	
Medications, n (%)				
Antibiotics	62 (0.1)	42 (0.8)	20 (0.0)	< 0.001
Corticosteroids	43,698 (38.0)	1,799 (32.4)	41,899 (38.3)	< 0.001
Biologics	3,078 (2.7)	88 (1.6)	2,990 (2.7)	< 0.001
Calcineurin inhibitors	959 (0.8)	43 (0.8)	916 (0.8)	0.612
Other immunosuppressants	4,024 (3.5)	62 (1.1)	3,962 (3.6)	< 0.001
Respiratory conditions, n (%)				
Pulmonary hypertension	9,611 (8.4)	394 (7.1)	9,217 (8.4)	< 0.001
Acute respiratory failure	17,880 (15.6)	465 (8.4)	17,415 (15.9)	< 0.001
Asthma	20,259 (17.6)	686 (12.4)	19,573 (17.9)	< 0.001
COPD	40,500 (35.2)	1,688 (30.4)	38,812 (35.5)	< 0.001
Pneumonia	29,785 (25.9)	1,110 (20.0)	28,675 (26.2)	< 0.001
Lung transplantation	312 (0.3)	46 (0.8)	266 (0.2)	< 0.001
URTI	25,908 (22.5)	1,052 (18.9)	24,856 (22.7)	< 0.001
LRTI	21,788 (19.0)	876 (15.8)	20,912 (19.1)	< 0.001
Lung cancer	4,598 (4.0)	75 (1.4)	4,523 (4.1)	< 0.001
Cystic fibrosis	217 (0.2)	3 (0.1)	214 (0.2)	0.018
Other clinical characteristics, n (%)				
Oxygen therapy use	21,343 (18.6)	776 (14.0)	20,567 (18.8)	< 0.001
Evidence of smoking	46,885 (40.8)	1,747 (31.5)	45,138 (41.3)	< 0.001
Pulmonary rehabilitation	773 (0.7)	42 (0.8)	731 (0.7)	0.435

Abbreviations: IPF– idiopathic pulmonary fibrosis; ILD– interstitial lung disease; SD– standard deviation; URTI– upper respiratory tract infection; LRTI– lower respiratory tract infection

Notes: ^1^States included in each region are as follows: **Northeast** (CT, MA, ME, NH, RI, VT, NJ, NY, PA); **Midwest** (IL, IN, MI, OH, WI, IA, KS, MN, MO, ND, NE, SD); **South** (DC, DE, FL, GA, MD, NC, SC, VA, WV, AL, KY, MS, TN, AR, LA, OK, TX); **West** (AZ, CO, ID, MT, NM, NV, UT, WY, AK, CA, HI, OR, WA)

^2^The identification period used only partial years in 2016 (October - December) and 2022 (January - July)

### Outcomes

The mean (SD) and median (IQR) follow-up time for all fibrosing ILD patients were 24 (19) and 19 (8–37) months, respectively. A longer mean follow-up time was observed among patients with IPF compared to those with non-IPF ILD (27 vs. 24 months, *p* < 0.001); 24% of patients with IPF and 18% of patients with non-IPF ILD ended follow-up due to death (*p* < 0.001). Among patients without evidence of oxygen therapy use during the pre-ILD baseline period, the incidence rate of initiating oxygen therapy after ILD diagnosis was 155.6 cases per 1,000 person-years (Supplementary Table S1).

**Fig. 2:**
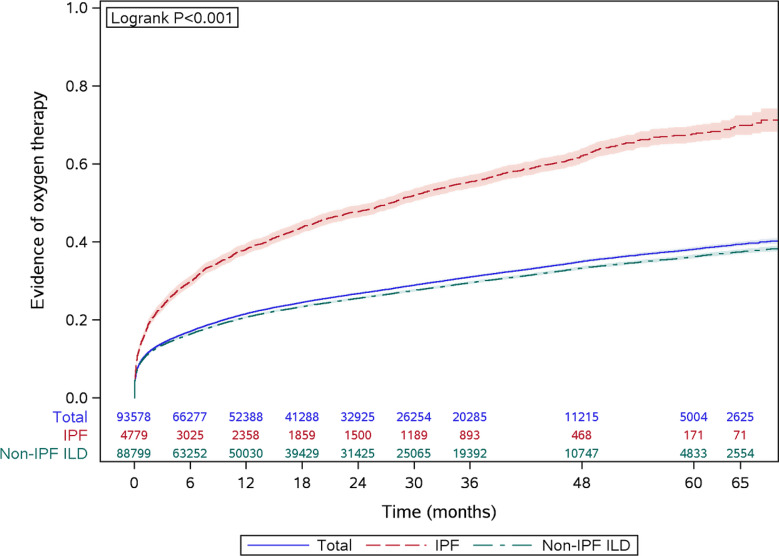
Kaplan-Meier analysis of time to initiation of oxygen therapy, among patients without baseline oxygen therapy use, stratified by ILD type. Notes: Log-rank test was used to assess equality of distributions

Figure 2 depicts the time to oxygen therapy initiation from the index date. The censor-adjusted proportion of patients initiating oxygen therapy increased over time since ILD diagnosis, and 38% of fibrosing ILD patients had evidence of initiating oxygen therapy during the follow-up period. Approximately 68% of patients with IPF and 36% of patients with non-IPF ILD initiated oxygen therapy within 60 months following initial ILD diagnosis (Fig. 2). A total of 11,931 patients (IPF *n* = 1,339; non-IPF ILD *n* = 10,592) had at least 12 months of follow-up after initiating oxygen therapy. Of these, 5,940 patients (49.8%) demonstrated sustained oxygen therapy use, with a mean (SD) of 11.4 (13.4) claims for oxygen therapy within the first 12 months of oxygen use. A higher proportion of patients with IPF exhibited sustained oxygen therapy use compared to non-IPF ILD (72.2% vs. 47.0%, *p* < 0.001).

**Fig. 3:**
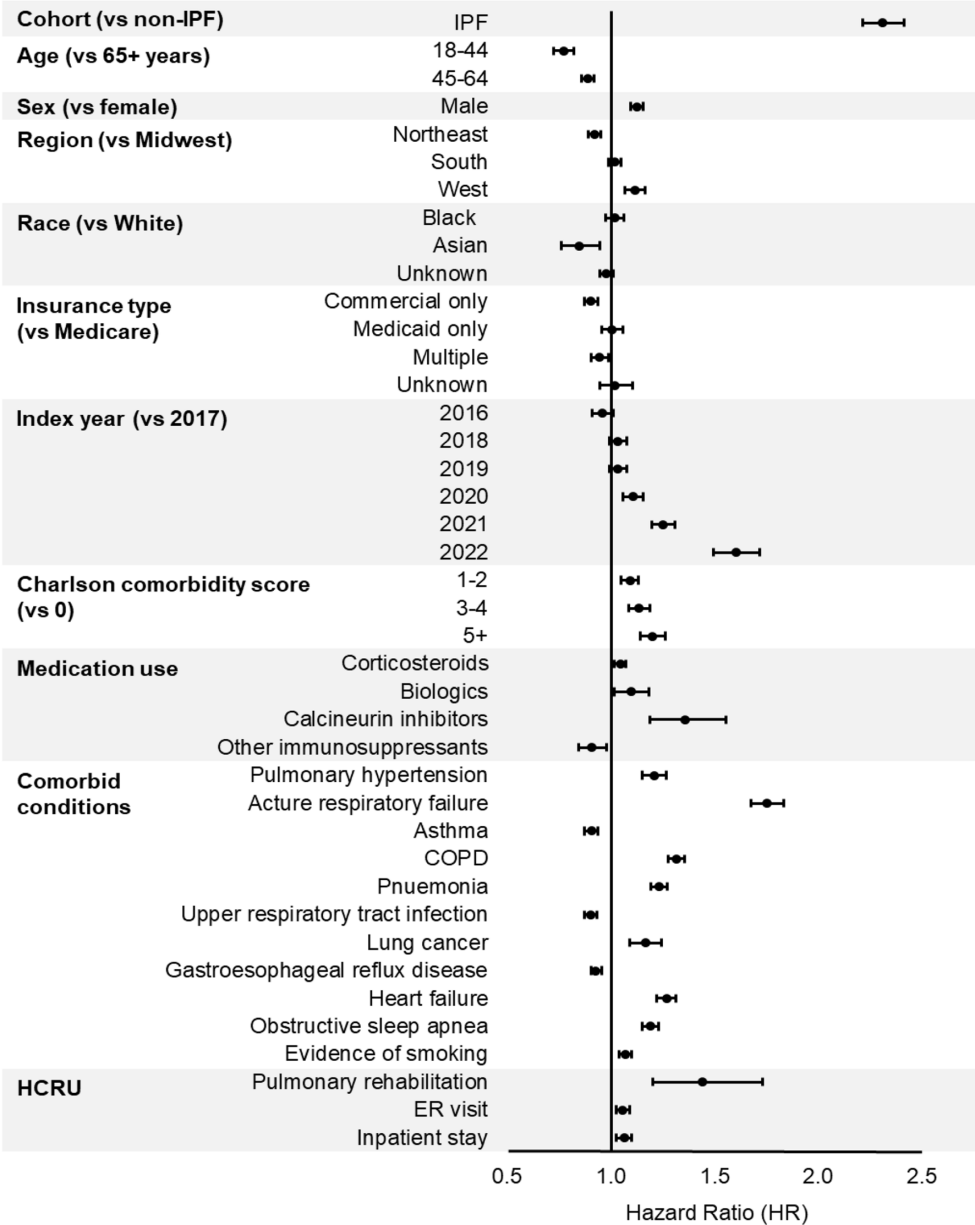
Cox proportional hazards model: factors associated with oxygen therapy initiation. Abbreviations: IPF– idiopathic pulmonary fibrosis; COPD– chronic obstructive pulmonary disease; HCRU– Healthcare resource utilization; ER– emergency room

The unadjusted hazard of oxygen therapy initiation among patients with IPF was 2.19 times the hazard among patients with non-IPF ILD (95% CI [2.10, 2.28], *p* < 0.001). After adjusting for demographic and baseline clinical characteristics, the hazard of oxygen therapy initiation among patients with IPF remained more than twice the hazard among those with non-IPF ILD (adjusted hazard ratio = 2.31; 95% CI [2.21, 2.41], *p* < 0.001) (Fig. 3). Furthermore, the hazard of oxygen therapy initiation increased with a higher CCI score and was higher among patients diagnosed with pulmonary hypertension, acute respiratory failure, COPD, pneumonia, heart failure, and those who achieved pulmonary rehabilitation. (Fig. 3).

## Discussion

To our knowledge, this is the first real-world evidence study to describe the initiation of and factors associated with initiation of supplemental oxygen therapy among patients with fibrosing ILD. The study findings demonstrate that a substantial proportion of patients initiate oxygen therapy following ILD diagnosis, with a higher proportion of patients with IPF initiating oxygen therapy compared to those with non-IPF ILD. Patients with higher CCI scores and comorbidities that affect lung function and respiratory capacity were more likely to initiate oxygen therapy.

In this study, nearly 40% of fibrosing ILD patients had evidence of oxygen therapy within the first 5 years following their ILD diagnosis. The sharpest increase in the incidence rate was observed during the first 12 months post-diagnosis, followed by a steady increase thereafter. A similar trend was observed in a previous study, which found that the majority of patients initiated oxygen therapy within the first year following their IPF diagnosis [15]. There are potential reasons explaining the increased use of oxygen therapy among patients with fibrosing ILD. Hypoxemia is a common clinical event among patients with fibrosing ILD. A prior study demonstrated that 5-year cumulative incidence of exertional and resting hypoxemia were 40.1% (95% CI [34.7%, 45.5%]) and 16.5% (95% CI [13.6%, 19.7%]), respectively [16]. Supplemental oxygen therapy has been conditionally recommended by multiple clinical guidelines based on weak evidence to treat hypoxemia and its related symptoms and complications, however the benefits of supplemental oxygen therapy for patients with ILD are uncertain [17–19]. The UK AMBOX trial demonstrated an improvement in health status, as determined by the King’S Brief Interstitial Lung Disease questionaire, however, clinical significance of improvement was unclear [20]. Consequently, recommendations may have heightened physician’s awareness of oxygen therapy and influenced their practice as the role of supplemental oxygen therapy for patients with fibrosing ILD stills warrants active investigation. However, it is important to note that these recommendations are primarily based on expert opinions and lack direct empirical evidence.

While the use of oxygen therapy is prevalent among patients with fibrosing ILD, it is crucial to delve deeper into the differential usage pattern based on the underlying causes of fibrosis. A prior study established that patients with IPF have a higher risk of developing hypoxemia compared to those with non-IPF ILD [23]. The increased risk may be attributed to the varying degrees of interstitial fibrosis and differences in disease progression. Our study corroborates these findings, as we observed a higher utilization of oxygen therapy among patients with IPF compared to those with non-IPF ILD. However, it is important to highlight that a subset of patients with non-IPF ILD may also progress and demonstrate a disease course similar to that of IPF [2, 24]. This study did not distinguish the incidence of oxygen therapy initiation among patients with non-IPF ILD based on their progression status or underlying ILD type. This is a significant area that warrants further exploration to better understand the correlation between disease progression or etiology and oxygen therapy initiation in non-IPF ILD patients.

In this study, increased oxygen therapy initiation was associated with higher CCI score and the presence of respiratory comorbidities. The association between high CCI score and oxygen therapy initiation suggests that patients with more severe comorbidity profiles are more likely to require oxygen therapy, possibly due to the additional strain these comorbidities place on their respiratory system. Notably, the presence of respiratory comorbidities (e.g., pulmonary hypertension, COPD, pneumonia, lung cancer) was positively associated with oxygen therapy initiation. Specifically, patients with acute respiratory failure were 75% more likely to initiate oxygen therapy than those without this entity. Our findings are consistent with existing data, which underscores the importance of comorbidity profile in managing ILD and its negative impact on burden and survival of patients with fibrosing ILD [4, 21–24]. 

The impact of supplemental oxygen on ILD symptom management and the long-term outcomes following the oxygen therapy initiation remain uncertain. Prior studies have demonstrated that oxygen therapy may improve exercise capacity and quality of life [20], and could potentially contribute to palliative care [31]. However, oxygen therapy may be burdensome for some patients [25]. Qualitative studies have demonstrated that initiation of oxygen therapy can often be perceived negatively by patients with fibrosing ILD, as it is seen as an indicator of disease progression and the beginning of a restricted lifestyle [26–28]. Consideration should also be given to practical and logistical barriers that may hinder the use of supplemental oxygen. Previous studies have identified gaps in patient education regarding ILD and oxygen therapy [28–30]. Furthermore, inadequate funding for oxygen therapy supplies and regular recurring costs may result in a substantial economic burden on patients with fibrosing ILD and suboptimal oxygen use [28, 31–33]. Therefore, further research on the impact and burden associated with supplemental oxygen therapy among patients with fibrosing ILD is essential to inform practice guidelines and adequate funding for the treatment of these patients.

This study used real-world data to provide insights into current trends in the use of oxygen therapy among patients with fibrosing ILD. However, there are several limitations to this study. First, oxygen therapy use was identified by the presence of codes on administrative claims. Due to lack of granularity in oxygen therapy codes, we could not assess the specific forms of oxygen therapy in clinical practice (e.g., nocturnal oxygen therapy, short-burst oxygen therapy), daily usage duration, and the type of hypoxemia for which the therapy was prescribed. A broad range of codes was used, including noninvasive respiratory ventilation systems, which was intended to capture a broad range of oxygen therapy use among patients with ILD. However, given the lack of studies directly supporting the respiratory ventilation systems for treatment of ILD, inclusion of these codes may result in misclassification bias. Second, there is no established definition for the sustained use of oxygen therapy for treatment of ILD. The definition of sustained oxygen therapy use in this study was derived from previous research conducted among COPD patients. However, COPD and ILD are distinct diseases with different pathophysiology, clinical manifestations, and disease progression. As such, the definition of sustained oxygen therapy use in COPD may not be entirely applicable to ILD patients. Third, all patients included in this study were continuously enrolled in a health plan during the study period; while this study sought to establish the incidence of oxygen therapy use among patients with ILD, it more closely measured the incidence among insured patients with ILD who were eligible for and consented to receiving oxygen therapy. Lastly, though IPF is one of the most common forms of fibrotic ILD, IPF represented only a small proportion of the ILD population identified in our study; given that ILD was defined based on diagnosis codes, incorrect coding of IPF diagnosis may contribute to misclassification of patients with IPF as having non-IPF ILD.

## Conclusion

The study findings demonstrate that a substantial proportion of patients initiate oxygen therapy following ILD diagnosis, particularly within the first 12 months after diagnosis. Oxygen therapy use is more pronounced in patients with IPF compared to those with non-IPF ILD, suggesting a potential association between the use of oxygen therapy and disease progression. Moreover, comorbidity burden, particularly respiratory diseases, was a key factor associated with the increased initiation of oxygen therapy. These findings underscore the need for improved understanding of outcomes following oxygen therapy initiation.

## Electronic supplementary material

Below is the link to the electronic supplementary material.

Supplementary Material 1

## Data Availability

No datasets were generated or analysed during the current study.
