# Supplementary material for: Supplemental oxygen therapy use among patients with fibrosing interstitial lung disease in the United States
Source: Respir Res. 2025 Feb 28;26:80. doi: 10.1186/s12931-025-03139-3 (PMC11871663; doi:10.1186/s12931-025-03139-3)
Supplement: Supplementary file 1 — Supplementary Material 1 [file 12931_2025_3139_MOESM1_ESM.docx]

**Supplementary Materials**

**Supplementary Table 1.** Codes used to identify oxygen therapy

| **Code** | **Code Type** | **Description** |
| --- | --- | --- |
| 94002 | CPT | Ventilation assist and management, initiation of pressure or volume preset ventilators for assisted or controlled breathing; hospital inpatient/observation, initial day |
| 94003 | CPT | Ventilation assist and management, initiation of pressure or volume preset ventilators for assisted or controlled breathing; hospital inpatient/observation, each subsequent day |
| 94004 | CPT | Ventilation assist and management, initiation of pressure or volume preset ventilators for assisted or controlled breathing; nursing facility, per day |
| 94005 | CPT | Home ventilator management care plan oversight of a patient (patient not present) in home, domiciliary or rest home (eg, assisted living) requiring review of status, review of laboratories and other studies and revision of orders and respiratory care plan (as appropriate), within a calendar month, 30 minutes or more |
| 94660 | CPT | Continuous positive airway pressure ventilation (CPAP), initiation and management |
| 94662 | CPT | Continuous negative pressure ventilation (CNP), initiation and management |
| 99504 | CPT | Home visit for mechanical ventilation care |
| A4483 | HCPCS | Moisture exchanger, disposable, for use with invasive mechanical ventilation |
| E0424 | HCPCS | Stationary compressed gaseous oxygen system, rental; includes container, contents, regulator, flowmeter, humidifier, nebulizer, cannula or mask, and tubing |
| E0425 | HCPCS | Stationary compressed gas system, purchase; includes regulator, flowmeter, humidifier, nebulizer, cannula or mask, and tubing |
| E0430 | HCPCS | Portable gaseous oxygen system, purchase; includes regulator, flowmeter, humidifier, cannula or mask, and tubing |
| E0431 | HCPCS | Portable gaseous oxygen system, rental; includes portable container, regulator, flowmeter, humidifier, cannula or mask, and tubing |
| E0433 | HCPCS | Portable liquid oxygen system, rental; home liquefier used to fill portable liquid oxygen containers, includes portable containers, regulator, flowmeter, humidifier, cannula or mask and tubing, with or without supply reservoir and contents gauge |
| E0434 | HCPCS | Portable liquid oxygen system, rental; includes portable container, supply reservoir, humidifier, flowmeter, refill adaptor, contents gauge, cannula or mask, and tubing |
| E0435 | HCPCS | Portable liquid oxygen system, purchase; includes portable container, supply reservoir, flowmeter, humidifier, contents gauge, cannula or mask, tubing and refill adaptor |
| E0439 | HCPCS | Stationary liquid oxygen system, rental; includes container, contents, regulator, flowmeter, humidifier, nebulizer, cannula or mask, & tubing |
| E0440 | HCPCS | Stationary liquid oxygen system, purchase; includes use of reservoir, contents indicator, regulator, flowmeter, humidifier, nebulizer, cannula or mask, and tubing |
| E0441 | HCPCS | Stationary oxygen contents, gaseous, 1 month's supply = 1 unit |
| E0442 | HCPCS | Stationary oxygen contents, liquid, 1 month's supply = 1 unit |
| E0443 | HCPCS | Portable oxygen contents, gaseous, 1 month's supply = 1 unit |
| E0444 | HCPCS | Portable oxygen contents, liquid, 1 month's supply = 1 unit |
| E0450 | HCPCS | Volume control ventilator, without pressure support mode, may include pressure control mode, used with invasive interface (e.g., tracheostomy tube) |
| E0460 | HCPCS | Negative pressure ventilator; portable or stationary |
| E0461 | HCPCS | Volume control ventilator, without pressure support mode, may include pressure control mode, used with noninvasive interface (e.g., mask) |
| E0463 | HCPCS | Pressure support ventilator with volume control mode, may include pressure control mode, used with invasive interface (e.g., tracheostomy tube) |
| E0464 | HCPCS | Pressure support ventilator with volume control mode, may include pressure control mode, used with noninvasive interface (e.g., mask) |
| E0465 | HCPCS | Home ventilator, any type, used with invasive interface, (e.g., tracheostomy tube) |
| E0466 | HCPCS | Home ventilator, any type, used with non-invasive interface, (e.g., mask, chest shell) |
| E0467 | HCPCS | Home ventilator, multi-function respiratory device, also performs any or all of the additional functions of oxygen concentration, drug nebulization, aspiration, and cough stimulation, includes all accessories, components and supplies for all functions |
| E0470 | HCPCS | Respiratory assist device, bi-level pressure capability, without backup rate feature, used with noninvasive interface, e.g., nasal or facial mask (intermittent assist device with continuous positive airway pressure device) |
| E0471 | HCPCS | Respiratory assist device, bi-level pressure capability, with back-up rate feature, used with noninvasive interface, e.g., nasal or facial mask (intermittent assist device with continuous positive airway pressure device) |
| E0472 | HCPCS | Respiratory assist device, bi-level pressure capability, with backup rate feature, used with invasive interface, e.g., tracheostomy tube (intermittent assist device with continuous positive airway pressure device) |
| E0481 | HCPCS | Intrapulmonary percussive ventilation system and related accessories |
| E0550 | HCPCS | Humidifier, durable for extensive supplemental humidification during ippb treatments or oxygen delivery |
| E0560 | HCPCS | Humidifier, durable for supplemental humidification during ippb treatment or oxygen delivery |
| E1390 | HCPCS | Oxygen concentrator, single delivery port, capable of delivering 85 percent or greater oxygen concentration at the prescribed flow rate |
| E1391 | HCPCS | Oxygen concentrator, dual delivery port, capable of delivering 85 percent or greater oxygen concentration at the prescribed flow rate, each |
| E1392 | HCPCS | Portable oxygen concentrator, rental |
| E1405 | HCPCS | Oxygen and water vapor enriching system with heated delivery |
| E1406 | HCPCS | Oxygen and water vapor enriching system without heated delivery |
| K0738 | HCPCS | Portable gaseous oxygen system, rental; home compressor used to fill portable oxygen cylinders; includes portable containers, regulator, flowmeter, humidifier, cannula or mask, and tubing |
| K0741 | HCPCS | Portable gaseous oxygen system, rental, includes portable container, regulator, flowmeter, humidifier, cannula or mask, and tubing, for cluster headaches |
| K0742 | HCPCS | Portable oxygen contents, gaseous, 1 month's supply = 1 unit, for cluster headaches, for initial months supply or to replace used contents |
| S8120 | HCPCS | Oxygen contents, gaseous, 1 unit equals 1 cubic foot |
| S8121 | HCPCS | Oxygen contents, liquid, 1 unit equals 1 pound |
| Z9911 | ICD-10 Dx | Dependence on respirator [ventilator] status |
| 5A09357 | ICD-10 Proc | Assistance with Respiratory Ventilation, Less than 24 Consecutive Hours, Continuous Positive Airway Pressure |
| 5A09358 | ICD-10 Proc | Assistance with Respiratory Ventilation, Less than 24 Consecutive Hours, Intermittent Positive Airway Pressure |
| 5A09359 | ICD-10 Proc | Assistance with Respiratory Ventilation, Less than 24 Consecutive Hours, Continuous Negative Airway Pressure |
| 5A0935A | ICD-10 Proc | Assistance with Respiratory Ventilation, Less than 24 Consecutive Hours, High Nasal Flow/Velocity |
| 5A0935B | ICD-10 Proc | Assistance with Respiratory Ventilation, Less than 24 Consecutive Hours, Intermittent Negative Airway Pressure |
| 5A0935Z | ICD-10 Proc | Assistance with Respiratory Ventilation, Less than 24 Consecutive Hours |
| 5A09457 | ICD-10 Proc | Assistance with Respiratory Ventilation, 24-96 Consecutive Hours, Continuous Positive Airway Pressure |
| 5A09458 | ICD-10 Proc | Assistance with Respiratory Ventilation, 24-96 Consecutive Hours, Intermittent Positive Airway Pressure |
| 5A09459 | ICD-10 Proc | Assistance with Respiratory Ventilation, 24-96 Consecutive Hours, Continuous Negative Airway Pressure |
| 5A0945A | ICD-10 Proc | Assistance with Respiratory Ventilation, 24-96 Consecutive Hours, High Nasal Flow/Velocity |
| 5A0945B | ICD-10 Proc | Assistance with Respiratory Ventilation, 24-96 Consecutive Hours, Intermittent Negative Airway Pressure |
| 5A0945Z | ICD-10 Proc | Assistance with Respiratory Ventilation, 24-96 Consecutive Hours |
| 5A09557 | ICD-10 Proc | Assistance with Respiratory Ventilation, Greater than 96 Consecutive Hours, Continuous Positive Airway Pressure |
| 5A09558 | ICD-10 Proc | Assistance with Respiratory Ventilation, Greater than 96 Consecutive Hours, Intermittent Positive Airway Pressure |
| 5A09559 | ICD-10 Proc | Assistance with Respiratory Ventilation, Greater than 96 Consecutive Hours, Continuous Negative Airway Pressure |
| 5A0955A | ICD-10 Proc | Assistance with Respiratory Ventilation, Greater than 96 Consecutive Hours, High Nasal Flow/Velocity |
| 5A0955B | ICD-10 Proc | Assistance with Respiratory Ventilation, Greater than 96 Consecutive Hours, Intermittent Negative Airway Pressure |
| 5A0955Z | ICD-10 Proc | Assistance with Respiratory Ventilation, Greater than 96 Consecutive Hours |
| 5A19054 | ICD-10 Proc | Respiratory Ventilation, Single, Nonmechanical |
| 5A1935Z | ICD-10 Proc | Respiratory Ventilation, Less than 24 Consecutive Hours |
| 5A1945Z | ICD-10 Proc | Respiratory Ventilation, 24-96 Consecutive Hours |
| 5A1955Z | ICD-10 Proc | Respiratory Ventilation, Greater than 96 Consecutive Hours |
| Z9981 | ICD-10 Dx | Dependence on supplemental oxygen |
| 0277 | Revenue Code | Medical/surgical supplies: take home oxygen |
| 0600 | Revenue Code | Home health-oxygen |
| 0601 | Revenue Code | Home Health Oxygen - Stat/Equip/Supply or contents |
| 0602 | Revenue Code | Home Health Oxygen - Stat/Equip/Supply Under 1 LPM |
| 0603 | Revenue Code | Home Health Oxygen - Stat/Equip Over 4 LPM |
| 0604 | Revenue Code | Home Health Oxygen - Portable Add-on |
| 0609 | Revenue Code | Home Health Oxygen - Other |

**Supplementary Table 2. Pre-ILD baseline all-cause healthcare resource utilization and healthcare costs**

|  | Total (N=114,921) | IPF (N=5,555) | Non-IPF ILD (N=109,366) | p-value |
| --- | --- | --- | --- | --- |
| All-cause healthcare resource utilization, n (%) | | | | |
| Ambulatory visit | 110,441 (96.1) | 5,277 (95.0) | 105,164 (96.2) | <0.001 |
| Office visit | 107,018 (93.1) | 5,103 (91.9) | 101,915 (93.2) | <0.001 |
| Outpatient visit | 89,029 (77.5) | 3,946 (71.0) | 85,083 (77.8) | <0.001 |
| Emergency room visit | 64,679 (56.3) | 2,136 (38.5) | 62,543 (57.2) | <0.001 |
| Inpatient stay | 31,154 (27.1) | 891 (16.0) | 30,263 (27.7) | <0.001 |
| Pharmacy use | 99,217 (86.3) | 4,582 (82.5) | 94,635 (86.5) | <0.001 |
| All-cause healthcare resource utilization counts, mean (SD) | | | | |
| Ambulatory visits | 26.5 (29.6) | 19.6 (21.1) | 26.9 (29.9) | <0.001 |
| Office visits | 16.2 (18.2) | 13.6 (16.1) | 16.3 (18.3) | <0.001 |
| Outpatient visits | 10.6 (20.7) | 6.2 (11.0) | 10.8 (21.0) | <0.001 |
| Emergency room visits | 1.9 (5.3) | 0.9 (2.4) | 2.0 (5.4) | <0.001 |
| Inpatient stays | 0.5 (1.3) | 0.2 (0.7) | 0.5 (1.3) | <0.001 |
| Inpatient days, among all patients | 6.3 (25.0) | 2.5 (22.0) | 6.5 (26.1) | <0.001 |
| Inpatient days among patients with ≥1 inpatient stay | 23.1 (45.8) | 15.8 (53.1) | 23.3 (45.5) | <0.001 |
| Pharmacy fills | 40.0 (45.9) | 29.5 (35.1) | 40.5 (46.3) | <0.001 |
| All-cause healthcare costs, mean (SD) | | | | |
| Total costs  (medical + pharmacy) | $56,948 ($109,105) | $27,364 ($51,630) | $58,450 ($111,024) | <0.001 |
| Medical costs | $50,360 ($98,124) | $23,081 ($47,678) | $51,746 ($99,811) | <0.001 |
| Ambulatory | $15,094 ($39,948) | $8,277  ($17,956) | $15,441 ($40,719) | <0.001 |
| Office visits | $4,748  ($15,823) | $3,221  ($7,599) | $4,826  ($16,126) | <0.001 |
| Outpatient visits | $10,347 ($35,384) | $5,056  ($15,383) | $10,615 ($36,085) | <0.001 |
| Emergency room visits | $2,560  ($8,424) | $988  ($3,018) | $2,640  ($8,601) | <0.001 |
| Inpatient stays | $23,012 ($73,027) | $8,577  ($33,588) | $23,745 ($74,401) | <0.001 |
| Other medical costs | $9,694  ($27,648) | $5,239  ($17,481) | $9,920  ($28,048) | <0.001 |
| Pharmacy costs | $6,588  ($40,055) | $4,283  ($15,987) | $6,705  ($40,898) | <0.001 |

**Notes:** Costs are adjusted using the annual medical care component of the Consumer Price Index (CPI) to reflect inflation to year 2022.

US Department of Labor, Bureau of Labor Statistics. Consumer Price Index. Medical Care. Pearson chi-square test was used for binary measures; two-sample t-test used for continuous meausres.

| **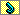Supplementary Table 3.** Unadjusted Incidence of Oxygen Therapy Use Among Patients Without Oxygen Therapy Use in the Pre-ILD Baseline Period | | | | | | | | | | | | |
| --- | --- | --- | --- | --- | --- | --- | --- | --- | --- | --- | --- | --- |
|  |  |  |  |  |  |  |  |  |  |  |  |  |
| **Oxygen therapy use** | **Incidence Rates** | | | | | | | | | **Incidence Rate Ratio** | | |
|  | **Total (N=93,578)** | | | **IPF (N=4,779)** | | | **Non-IPF ILD (N=88,799)** | | | **IPF vs Non-IPF ILD** | |  |
|  | **Events** | **Person-time (years)** | **Rate** | **Events** | **Person-time (years)** | **Rate** | **Events** | **Person-time (years)** | **Rate** | **Ratio** | **p-value** |  |
| Evidence of oxygen therapy during variable follow-up | 24,686 | 158,616 | 0.1556 | 2,416 | 7,114 | 0.3396 | 22,270 | 151,502 | 0.1470 | 2.3104 | <0.001 |  |

**Notes:** An exact binomial distribution was used to assess significance of incidence rate ratio
